# Clinicopathological Significance of MicroRNA-214 in Gastric Cancer and Its Effect on Cell Biological Behaviour

**DOI:** 10.1371/journal.pone.0091307

**Published:** 2014-03-10

**Authors:** Ya-Wen Wang, Duan-Bo Shi, Xu Chen, Chao Gao, Peng Gao

**Affiliations:** Department of Pathology, School of Medicine, Shandong University, Jinan, P.R. China; University of Hong Kong, Hong Kong

## Abstract

Accumulating evidence indicates that numerous microRNAs are involved in the tumorigenesis and progression of gastric cancer, while the clinical significance of microRNA-214 in gastric cancer is poorly understood and the exact role of microRNA-214 in gastric cancer remains obscure. In the present study, expression levels of microRNA-214 in 80 gastric carcinoma tissues, 18 nontumourous gastric tissues, and 4 types of gastric cancer cell lines were quantified by reverse transcription followed by real-time quantitative polymerase chain reaction (RT-qPCR), and the relationship between microRNA-214 expression and cliniopathological characteristics including prognosis was explored. To investigate the potential role of microRNA-214 in gastric cancer cell biological behaviour, we performed cell proliferation, apoptosis, migration and invasion assays in four gastric cancer cell lines and an immortalized gastric cell line *in vitro*. Our results showed that microRNA-214 was dramatically downregulated in gastric cancer tissues and gastric cancer cell lines, compared with nontumourous gastric tissues. Stepwise downregulation of microRNA-214 expression was observed among nontumourous gastric mucosa, nonmetastasis gastric cancer tissues, and metastasis gastric cancer tissues. The expression of microRNA-214 was significantly inversely correlated with lymph node metastasis and tumour size but had no correlation with the patient's prognosis. Ectopic expression of microRNA-214 could inhibit cell migration and invasion ability in SGC7901 and MKN45 gastric cancer cells. And knockdown of microRNA-214 significantly facilitated cell proliferation, migration and invasion in a cell-specific manner in MKN28, BGC823 and GES-1 cells. Colony stimulating factor 1 (CSF1) was identified as a target gene of microRNA-214. In summary, our data demonstrated that microRNA-214 is a promising novel biomarker for lymph node metastasis in patients with gastric cancer. And we identified that downregulation of microRNA-214 may regulate the proliferation, invasion and migration of gastric cancer cells by directly targeting CSF1.

## Introduction

Gastric cancer (GC) is the fourth most common cancer and the second leading cause of cancer mortality worldwide [1]. Despite considerable studies on the tumourigenesis and progression of GC, the pathogenesis of this complex disease is poorly understood. Thus, it is of vital clinical value to identify and characterize the precise molecular mechanism involved in the development and progression of gastric carcinoma.

Apart from conventional genetic and epigenetic alteration of protein-coding oncogenes and tumour-suppressor genes in the carcinogenesis of GC, nonprotein-coding RNAs, especially microRNAs (miRNAs), have emerged as a new player to shed light on the mechanism of GC development [2]. MiRNAs are endogenous 19–25 nt noncoding RNAs that negatively regulate protein expression by promoting mRNA degradation or repressing protein translation, through interaction with the 3′-UTR of target mRNAs. A growing number of miRNAs have been reported to participate in carcinogenesis and development of human cancers, including GC [2]–[4]. These miRNAs are usually dysregulated and function either as tumour suppressors or oncogenes in the initiation and progression of human carcinomas. For instance, Tsukamoto et al. have shown that miR-375 is downregulated in gastric carcinoma and exerts its proapoptotic effect through downregulating PDK1, a kinase that phosphorylates Akt, and in turn, suppresses the PI3K/Akt pathway [5]. While miR-21 has been found to promote tumour proliferation and invasion in GC by negatively regulating important tumour suppressors such as PTEN, PDCD4, and RECK, and then confer GC cells with increased invasiveness and the ability to avoid anoikis [6]–[8]. Previously, we have found that miR-145 was downregulated in manifold human cancer cells and suppressed the invasion-metastasis cascade in GC by inhibiting N-cadherin protein translation [9], [10].

At present, the clinical significance of microRNA-214 (miR-214) in the prognosis of patients with GC is poorly understood, and the exact role of miR-214 in GC remains unclear. Here, we investigated the association between miR-214 expression and cliniopathological parameters as well as assessed the effect of miR-214 on biological behaviours including cell proliferation, apoptosis, migration and invasion of GC cells.

## Materials and Methods

### Tissue samples

Tissue samples were prepared in a similar manner as described previously [9]. Briefly, 80 samples (from 65 males, 15 females; 58.3±17.49 and 61.5±9.162 years old, respectively) of GC tissues were obtained from patients who underwent surgical resection at Qi Lu Hospital of Shandong University from 2004 to 2006. Nontumourous gastric mucosa more than 3 cm away from tumours was randomly selected from 18 of these patients and used as controls. None of the patients received preoperative treatment, such as radiation therapy or chemotherapy. Specimens were typed histologically according to Lauren's and the World Health Organization (WHO) 's classifications (IARC Press, Lyon, 2000), and categorized according to the UICC 2002 TNM classification.

### Ethics statement

The study was approved by the Ethics Committee of School of Medicine, Shandong University, Shandong, China (approval code: 201101015). We obtained written informed consent from all participants involved in our study.

### Cell culture

Four types of human GC cell lines were obtained from the American Type Culture Collection (MKN28 and MKN45, Manassas, VA, USA) and the Shanghai Cancer Institute (BGC823 and SGC7901, Shanghai, China). The immortalized gastric mucosal epithelial cell line GES-1 was obtained from Beijing ComWin Biotech Co., Ltd. (Beijing, China). The cells were maintained in RPMI 1640 culture medium supplemented with 10% fetal bovine serum (FBS) in a humidified cell incubator with an atmosphere of 5% CO_2_ at 37°C.

### MiRNA extraction

Using a miRNeasy FFPE Kit (Bioteke, Beijing, China), we isolated miRNA from paraffin-embedded tissues according to the manufacturer's instructions. Total RNA of cell lines was extracted using Trizol reagent (TaKaRa, Dalian, China) following the manufacturer's protocol. The quality and quantity of the RNA samples were assessed by standard spectrophotometric methods (BioPhotometer plus; Eppendorf, Hamburg, Germany) and diluted to 2 ng/µl for RT-qPCR analysis.

### Reverse transcription followed by real-time quantitative polymerase chain reaction (RT-qPCR)

MiRNA expression levels were quantitated using a SYBR Primescript miRNA RT PCR Kit (TaKaRa, Dalian, China) according to the manufacturer's instructions with a Bio-Rad CFX™ 96 C1000 Real-Time system. Briefly, the RNA samples were converted to cDNA by a One-step Primescript miRNAcDNA Synthesis Kit (TaKaRa, Dalian, China), followed by real-time qPCR and normalized using U6 small nuclear RNA (RNU6B) by the 2^−ΔCT^ method. Primers for miR-214 and U6 were from GeneCopoeia (HmiRQP0320, HmiRQP9001). All reactions were run in duplicate.

### Cell transfection

Exponentially growing cells (1.5×10^5^) were seeded in 12-well plates 12 h before transfection and were transfected with 30 nM miR-214 precursor (miR-214), anti-miR-214 inhibitor (miR-214 inhibitor), or the negative control (Ambion, Austin, TX, USA) using the X-tremeGENE transfection reagent (Roche Applied Science, Indianapolis, IN, USA) according to the manufacturer's instructions. Transfection efficiency was monitored by RT-qPCR at 24, 48, and 72 h after transfection.

For stable expression of miR-214, SGC7901 and MKN45 cells were transfected with lentivirus miR-214-expressing vector LV3-hsa-miR-214 or a negative control LV3NC, which has GFP as a marker protein, according to the manufacturer's protocol (GenePharma, Shanghai, China). Three days after transfection, the expression of GFP protein was monitored under fluorescence microscope. The transfected cells were screened with puromycin (1.5 µg/mL) for the ones stably expressing miR-214.

### Cell proliferation assay

After transfection, cells were trypsinised, counted, and seeded onto 96-well plates at a density of 5×10^3^ cells/well. And then cell proliferation was measured using the EdU proliferation assay as previously reported [11]. Briefly, 24 h after transfection, cells were cultured in triplicate at 5×10^3^ cells per well in 96-well plates the day before EdU incubation (Ribobio, Guangzhou, China). After EdU labeling, the cells were treated with 100 µL of 1× Apollo reaction cocktail, stained with 100 µL of Hoechst 33342 (5 µg/mL) and visualized under a fluorescence microscope (Olympus, Japan). The percentage of EdU positive cells was defined as the proliferation rate. Data were obtained from three independent experiments and presented as means ± standard deviation (SD).

### Cell apoptosis assay

Three days after transfection of miR-214 precursor or inhibitor, cells were collected and stained using the Annexin V-FITC/PI Apoptosis Detection Kit (BestBio, Shanghai, China) as previously described [12]. In brief, cells were trypsinized, collected and then stained using the Annexin V-FITC/PI Apoptosis Detection Kit following the manufacturer's instructions. After incubation with Annexin V-FITC and PI, the apoptotic cells were immediately analyzed by flow cytometry. Early apoptotic cells were defined as the population that was PI negative and Annexin V-FITC positive, while late apoptotic cells were PI positive and Annexin V-FITC positive. The total apoptotic rate was calculated as the early apoptotic rate plus the late apoptotic rate. Annexin V-PE/7-AAD Apoptosis Detection Kit (KeyGEN, Nanjing, China) was used for the apoptosis assay of lentivirus vector transfected cells, similar to the above protocols. Each experiment was performed in triplicate, and data were presented as means ± SD.

### Cell migration and invasion assays

Cell migration and invasion assays were performed as previously described [13]. Migration assay was conducted with Transwell inserts with 8.0 mm pore size membrane (24-well format, Corning, New York, USA). To measure invasion ability of GC cells, the previously mentioned inserts were pre-coated with Matrigel matrix (BD Science, Sparks, MD, USA). The cells (1×10^5^) were resuspended in serum-free medium and seeded to the upper chamber. The lower chambers were filled with complete culture medium containing 10% FBS. After incubation at 37°C for 24 h, the migrated cells present on the lower side of the membrane were fixed, stained and counted. Each experiment was performed in triplicate.

### Luciferase assay

Dual-luciferase assays were performed as previously described [9]. The 3′-UTR fragments of CSF1 gene containing the miR-214 binding site was amplified by PCR from MKN45 cell RNA using the primers in Table S1, and inserted into the Xba1 site of pmirGLO miRNA target expression vector (Promega, San Lius Obispo, CA, USA). The resulting vector was named pmirGLO-CSF1. For the luciferase reporter assay, MKN45 and BGC823 cells were seeded in a 12-well plate the day before transfection. The cells were co-transfected with 30 nM of miR-214 precursor or miR-214 inhibitor, negative control and 30 ng pmirGLO-CSF1 using X-tremeGENE transfection reagent (Roche Applied Science). After 48 h transfection, luciferase activity was measured using the dual luciferase assay system (Promega) and normalized to Renilla luciferase activity. Each experiment was performed in triplicate.

### Western blot

At 48 h after transfection with miR-214 precursor or inhibitor, cells were subjected to western blot analysis as described previously [19]. Briefly, protein was extracted from cells or tissues. Lysates were resolved by electrophoresis, transferred to nitrocellulose membranes and blotted with antibodies against CSF1 (1∶1000, Epitomics) or β-actin (1∶1000, Santa). Data were obtained from three independent experiments.

### Statistical analysis

Analyses were performed using the statistical package Prism 5 software (GraphPad Software, San Diego, CA, USA). Differences were analyzed with the Student's *t*-test between two groups or with one-way ANOVA among three groups. The correlation between miR-214 expression and tumour size in primary GC was calculated by Spearman's correlation. In the survival curve, data were analyzed by Log-rank Test. To determine to which extent the expression of miR-214 could efficiently separate different clinical subsettings, receiver operating characteristic (ROC) curve analysis was constructed and the area under the curve (AUC) was calculated to assess the ability of miR-214 expression to differentiate between cancer cases and nontumourous cases, and to distinguish metastatic tissues and nonmetastatic tissues. *P*-values less than 0.05 were considered statistically significant.

## Results

### MiR-214 is downregulated in gastric carcinoma tissues and four GC cell lines, compared with nontumourous gastric mucosa

**Figure 1:**
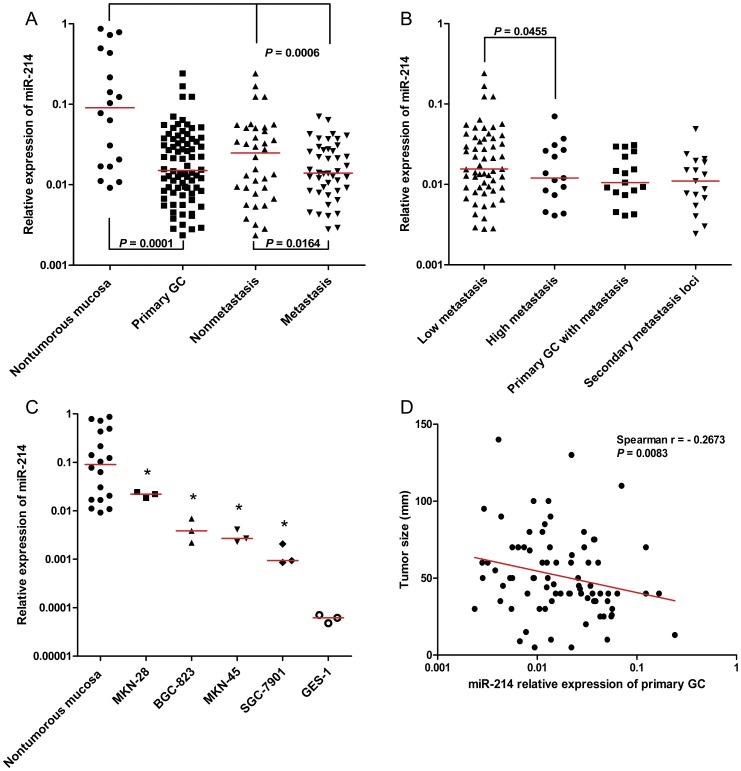
Expression of miR-214 in different samples and its association with tumour size. (A) In comparison with 18 nontumourous gastric mucosa, miR-214 was significantly downregulated in 80 primary gastric tissues (*P* = 0.0001). MiR-214 expression was e en lower in primary gastric tissues with metastasis (Metastasis) than primary gastric tissues without metastasis (Nonmetastais). (B) Primary gastric tissues were further divided into a low-metastasis group and a high-metastasis group according to the number of lymph node metastasis. (The cutoff was set as six, which is a threshold to distinguish N0∼N1 and N2∼N3 in TNM stage (UICC, 2002). MiR-214 was dramatically reduced in high-metastasis group compared to low-metastasis group (*P* = 0.0455). (C) MiR-214 downregulation was validated in four gastric cell lines. Compared to well-moderately differentiated cell line MKN28, miR-214 was markedly attenuated in poorly differentiated cell line MKN45 and BGC823, and moderately-poorly differentiated and highly metastatic cell line SGC7901. However, we detected lower expression of miR-214 in GES-1, an immortalized gastric epithelial cell line, compared with four gastric cancer cells (^*^ *P*<0.05). (D) Association between miR-214 expression and tumour size in primary GC was calculated by Spearman's correlation. Our data suggested that miR-214 expression was inversely correlated with tumor size (Spearman r = −0.2673, *P* = 0.0083).

In comparison with 18 nontumourous gastric mucosa samples, miR-214 was significantly downregulated (approximately six-fold) in 80 primary gastric tissue samples (Figure 1A, *P* = 0.0001). The receiver operating characteristic (ROC) curves of miR-214 reflected strong separation between GC tissues and nontumourous tissues, with an area under the curve (AUC) of 0.7764 (Figure S1A, 95% confidence interval (CI), 0.6466–0.9062). Consistently, downregulation of miR-214 was validated in four gastric cell lines. As shown in Figure 1C, the miR-214 expression level in the GC cell lines MKN28, BGC823, MKN45, and SGC7901 was markedly attenuated compared with 18 nontumourous gastric tissue samples (*P*<0.05).

However, we detected even lower expression of miR-214 in GES-1, an immortalized gastric epithelial cell line, than in four GC cell lines (Figure 1C, *P*<0.05). We speculate the discordance may be due at least in part to the difference between clinical samples and cell lines, because a cell line, isolated from one patient with a certain disease, represents the miRNA expression signature of only one clinical sample and may changes during cell culture *in vitro*; in other words, tissue samples are much more like the human context than cell lines. Thus, we comment that miR-214 expression in human GC tissues was more representative and credible than that found in cell lines.

### Decreased miR-214 expression in GC is associated with lymph node metastasis and tumour size but has no correlation with patient prognosis

To determine the potential clinicopathological implications of altered miR-214 expression, we combined the qPCR results and clinical parameters. Correlations between the miR-214 expression level and clinicopathological characteristics of GC are summarized in Table 1. MiR-214 expression was inversely correlated with tumour size (Table 1, *t*-test, *P* = 0.0265; Figure 1D, Spearman r = −0.2673, *P* = 0.0083) and lymph node metastasis (Table 1, Figure1A, *t*-test, *P* = 0.0164). However, there was no significant difference in other clinicopathological features such as gender, distal metastasis, WHO histological classification, and Lauren's histological type between these two groups.

**Table 1:** Association between miR-214 expression and clinicopathological parameters in 80 primary gastric cancer samples.

Variable	Number	Median	*P*-value
**Age (y)**			
≤61	36	0.02127	0.037
>61	44	0.01265	
**Sex**			
Male	65	0.01543	0.0962
Female	15	0.01405	
**Clinical Stage**			
I	14	0.01343	0.2791
II	17	0.01222	
III	24	0.02409	
IV	25	0.01553	
**Tumour size (mm)**			
≤30	17	0.03673	0.0265
>30	63	0.01468	
**Lymph node metastasis**			
Metastasis	44	0.01391	0.0164
Nonmetastasis	36	0.02476	
**Distal metastasis**			
Positive	15	0.01553	0.4151
Negative	65	0.01534	
**WHO histological classification**			
Well-differentiated	4	0.02749	0.1054
Moderately differentiated	25	0.01053	
Poorly differentiated	41	0.01534	
Mucinous adenocarcinoma	10	0.02492	
**Lauren's classification**			
Intestinal type	23	0.01422	0.3567
Diffuse type	57	0.01553	
**Relapse status**			
Relapse group	47	0.01553	0.1512
Nonrelapse group	33	0.01278	
**Prognosis**			
Survival group	34	0.0131	0.1363
Death group	46	0.01634	
**Total**	80	0.01501	

Intriguingly, we found stepwise downregulation of miR-214 among nontumourous gastric mucosa, nonmetastasis tissues, and metastasis tissues (Figure 1A, one-way ANOVA, *P* = 0.0006). To evaluate miR-214 expression in GC as a new biomarker for lymph node metastasis, ROC curves were established. We observed clear separations between the patients with lymph node metastasis and those without lymph node metastasis, with an AUC of 0.5880 (Figure S1B, 95% CI, 0.4526–0.7166). The primary gastric tissues were further divided into a low-metastasis group and a high-metastasis group according to the number of lymph node metastasis (LNM): low metastasis was defined as cases with less than six LNM, and cases with more than six LNM were considered as high metastasis. As expected, miR-214 expression was dramatically decreased in the high-metastasis group compared to the low-metastasis group (Figure 1B, *P* = 0.045).

In accordance with the tissue sample data indicating the correlation between miR-214 expression and LNM, we found that, compared to the moderately well-differentiated cell line MKN28, miR-214 expression was markedly less in the poorly differentiated cell lines MKN45 and BGC823, and especially the metastatic cell line SGC7901 [14] (*P*<0.05).

However, our data demonstrated no significant difference of miR-214 expression in 18 primary gastric tissue samples and their secondary metastasis loci (Figure 1B, *P* = 0.2676). These results suggest that downregulation of miR-214 expression occurred in the early stage of LNM development and remained stable without further attenuation in the late stage of metastasis.

**Figure 2:**
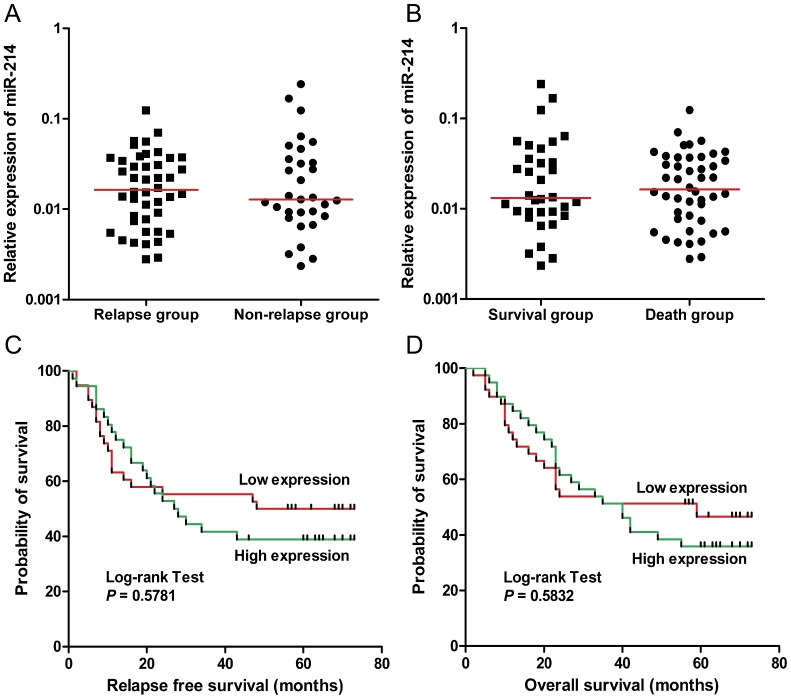
Effect of miR-214 expression on prognosis in patients with gastric cancer. (A) Tissue samples were divided into a release group and a nonrelease group according to the outcome of patients. Our data showed no significant difference in miR-214 expression between these two groups (*P*>0.05). (B) As in (A), except the clinical samples were classified as survival group and death group (*P*>0.05). (C, D) We divided the patients into high-expression and low-expression groups based upon the median level of miR-214 expression. The Kaplan–Meier survival curves showed no significant correlation between miR-214 expression and relapse free survival (hazard ratio (HR) 1.23, 95% confidence interval (CI) 0.6596, 2.285; *P* = 0.5781) or overall survival (HR 1.20, 95% CI 0.6667, 2.151; *P* = 0.5832), though there was a trend that high expression of miR-214 was associated with shorter relapse free survival (median survival: 28.00 months versus 74.50 months, for High expression and Low expression, respectively) or overall survival (median survival: 40.00 months versus 47.50 months, for High expression and Low expression, respectively).

To further explore the effect of miR-214 on the prognosis of patients, we analyzed the expression levels of miR-214 in cases with different relapse status and survival conditions. However, our data showed that there was no pronounced difference between the relapse group and the nonrelapse group, or between the survival group and the death group (Figure 2A–B, *t*-test, *P*>0.05). Of note, we divided the patients into high-expression and low-expression groups based upon the median level of miR-214 expression, and Kaplan–Meier survival curves showed no significant correlation between miR-214 expression and relapse-free survival (Figure 2C, hazard ratio (HR) 1.23, 95% confidence interval (CI) 0.6596-2.285; *P* = 0.5781) or overall survival (Figure 2D, HR 1.20, 95% CI 0.6667–2.151; *P* = 0.5832) in patients with GC.

### Effect of miR-214 on cell proliferation of GC cells

To monitor transfection efficiency, we determined miR-214 expression by RT-qPCR at 24, 48, and 72 h after transfection in the miR-214 precursor or inhibitor transfected cells and continuously detected miR-214 expression of lentivirus vectors treated cells for 4 weeks. As expected, transfection of the miR-214 precursor efficiently resulted in significant overexpression of miR-214 (Figure S2A–B, *P*<0.05) and miR-214 inhibitor strikingly reduced miR-214 expression in miR-214 inhibitor-transfected cells than the negative groups (Figure S3E–F, Figure S4 A, *P*<0.05). Also, we found that cells transfected with lentivirus vector LV3-hsa-miR-214 could lead to a 7 to 96-fold change of miR-214 expression in SGC7901 and MKN45 cells (Figure S3A-D, *P*<0.05), with 80%–90% cells expressing the GFP marker protein (Figure S3A, B).

**Figure 3:**
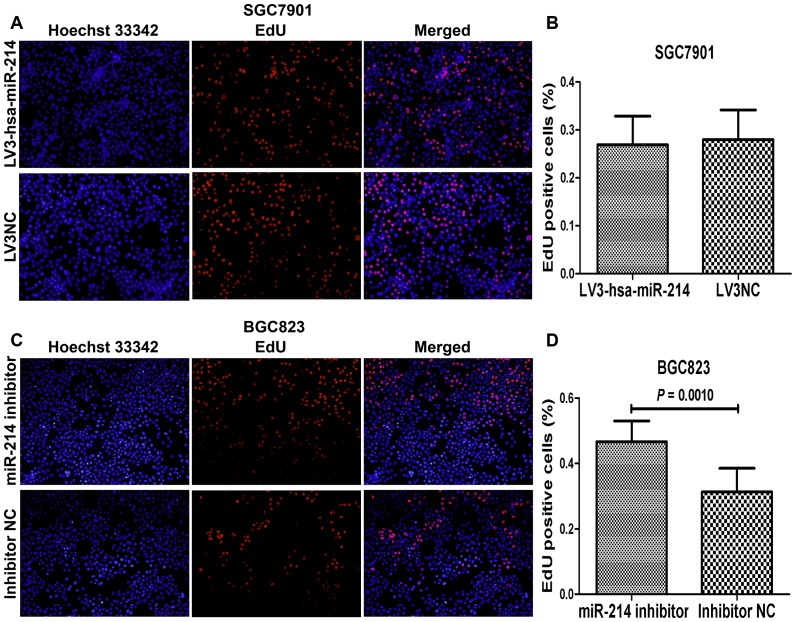
Effect of miR-214 on cell proliferation in SGC7901 and BGC823 cell lines using EdU assay. (A, C) Representative photographs after transfection with lentivirus miR-214-expressing vector in SGC7901 cells and miR-214 inhibitor in BGC823 cells (magnification 100×). (B, D) The percentage of EdU positive cells, calculated by EdU-labeled cell (red) number compared to total cell number (Hoechst-stained, blue), was defined as proliferation rate. The data showed no significant difference of the proliferation rate between LV3-hsa-miR-214-transfected group and the negative control group in SGC7901 cells (*P*>0.05). While we found that miR-214 inhibitor transfection could result in a strikingly increase proliferation ability of BGC823 cells (*P* = 0.0010).

To determine whether miR-214 could affect the proliferation of GC cells, EdU proliferation assay was used to detect cell growth ability. Our data showed that overexpression of miR-214 with lentiviurs vectors or miR-214 precursor did not influence cell growth of SGC7901 (Figure 3A-B, Figure S2C-D, P>0.05) and MKN45 cell lines (Figure S2E-F, Figure S5A-B, P>0.05). However, we found that downregualtion of miR-214 could promote the proliferation of BGC823 (Figure 3C-D, *P* = 0.0010) and GES-1 (Figure S4B-C, *P* = 0.0474), but not MKN28 cell line (Figure S5C-D, *P* = 0.0938), in a cell-specific manner.

### Role of miR-214 in cell migration and invasion of GC cells

**Figure 4:**
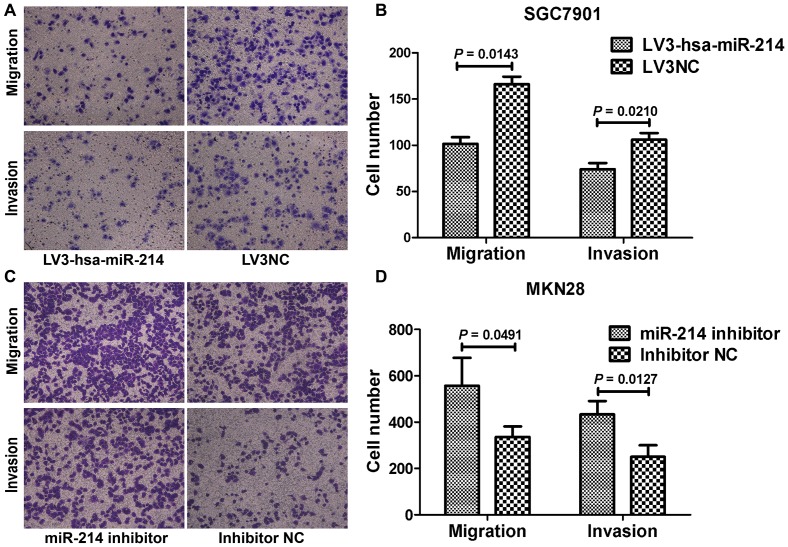
Influence of miR-214 on cell migration and invasion in SGC7901 and MKN28 cells. (A, C) Representative pictures of migration and invasion assays in SGC7901 and MKN28 cells (magnification 200×) are shown. (B, D) The migrated cells were counted by choosing five fields of each chamber randomly and calculating the average number. Our results showed that LV3-hsa-miR-214 transfection markedly decrease the migration and invasion ability of SGC7901 (*P* = 0.0143 and 0.0210, respectively). While knockdown of miR-214 with miR-214 inhibitor promotes cellular migration (*P* = 0.0491) and invasion (*P* = 0.0127) in MKN28 cells.

As miR-214 expression was inversely associated with lymph node metastasis, we were particularly interested in the ability miR-214 to affect cell migration and invasion. Our results indicated that SGC7901 and MKN45 cells transfected with LV3-hsa-miR-214 showed a significantly decreased migration and invasion capability, compared with the LV3NC treated cells (Figure 4A-B, Figure S6A-B, *P*<0.05). And we observed that downregualtion of miR-214 could facilitate the migration and invasion of MKN28 (Figure 4C–D, *P* = 0.0491 and *P* = 0.0127, respectively). Although knockdown of miR-214 did not affect the migration of GES-1 cells (Figure S4D–E, *P* = 0.0879), silencing of miR-214 led to a more than 40% increase in the invasive properties of these cells (*P* = 0.0046). However, our data showed that transfection of miR-214 precursor did not have a robust effect on cell migration and invasion in SGC7901 (Figure S2G-H, *P*>0.05) and MKN45 (Figure S2I-J, *P*>0.05) cells, compared to the respective negative controls.

### Influence of miR-214 on cell apoptosis of GC cells

**Figure 5:**
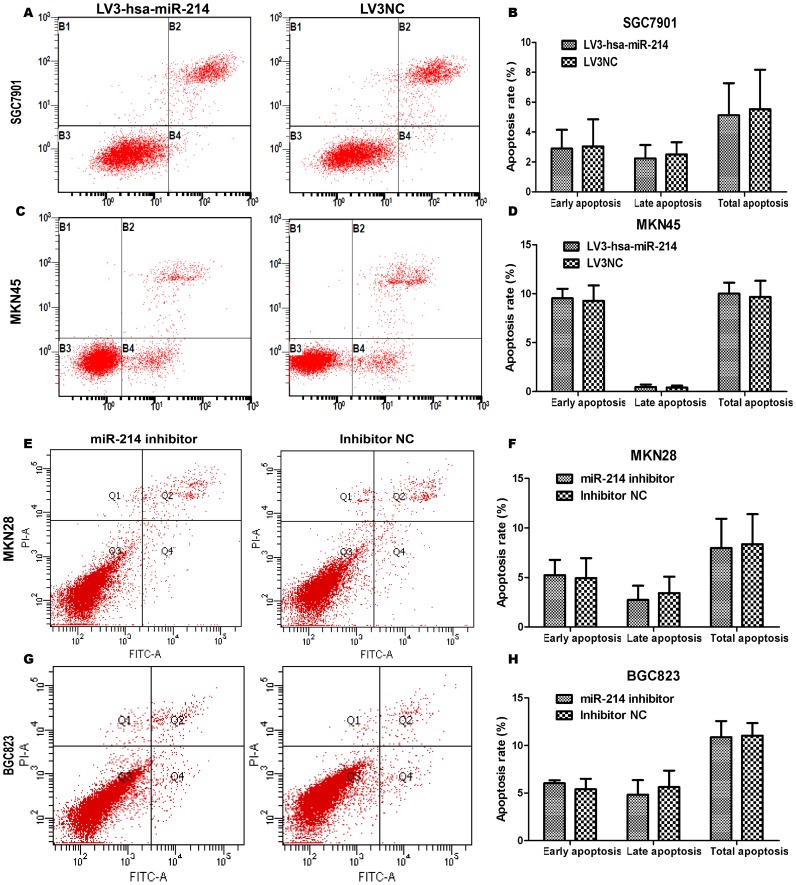
MiR-214 has no effect on cell apoptosis in four GC cells. (A, C, E, G) Cells were labeled with Annexin V-PE/7-AAD or Annexin V-FITC/PI, and analyzed by flow cytometry. All these figures are representative of three independent assays. Quadrant statistics: necrosis or mechanically-injured cells in upper left (UL), late apoptosis cells in upper right (UR), viable cells in lower left (LL) and early apoptosis cells in lower right (LR). (B, D, F, H) The percentage of early apoptosis cells, late apoptosis cells and total apoptosis cells were respectively compared between LV3hsa-miR-214 transfected group and NC group, or between miR-214 inhibitor-transfected group and inhibitor NC group. Our data demonstrated that LV3-hsa-miR-214 or miR-214 inhibitor has no effect on cell apoptosis in SGC7901, MKN45, MKN28 and BGC823 cells (*P*>0.05). Although we observed a trend of pro-apoptosis ability of miR-214 in MKN45 cell line, however, the difference was not statistically significant (*P* = 0.0950).

To examine the effect of miR-214 on cell apoptosis, we performed apoptosis assays using the Annexin V-FITC/PI staining method. Our results demonstrated that overexpression (miR-214 precursor and lentivirus vector) and knockdown of miR-214 could not affect cell apoptosis obviously compared with negative controls in four GC cell lines (Figure 5, Figure S2K-N, *P*>0.05) and immortalized gastric cell line GES-1 (data not shown). In fact, we found a tendency of pro-apoptosis ability of miR-214 precursor in MKN45 cell line (Figure S2M-N, *P* = 0.0606), however, the difference was not statistically significant.

### MiR-214 directly targets and down-regulates CSF1 in gastric cancer cells

**Figure 6:**
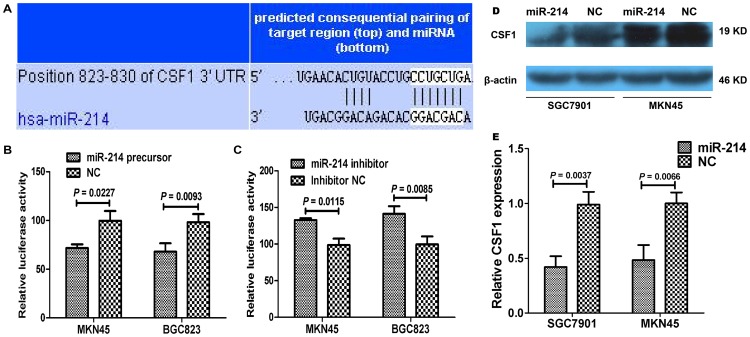
CSF1 is a direct target of miR-214. (A) The complementary sequences of the CSF1 mRNA 3′-UTR are shown with the miR-214 sequence. (B) Luciferase activity in MKN45 and BGC823 cells transfected with miR-214 and pmirGLO-CSF1 was significantly deceased compared with the negative controls (*P* = 0.0227 and 0.0093, respectively). (C) After miR-214 inhibitor was transfected, luciferase activity was dramatically increased in MKN45 and BGC823 cells compared with the controls (*P* = 0.0115 and 0.0085, respectively). (D) The effect of miR-214 on CSF1 levels were tested in GC cell lysates by western blot. (E). Our data showed that the relative CSF1 expression was significantly decreased in cells transfected with miR-214 precrusor as compared to the cells transfected with negative control (*P* = 0.0037 and 0.0066, respectively).

To identify targets of miR-214, we used TargetScan algorithm to predict miR-214 targets in human gastric cancer. Among the numerous possible candidates, we picked out the ones overexpressed in cancers and proliferation- and metastasis-associated genes, including NOTCH2, FGFR1, CSF1 (Figure 6A), AGAP2, CREB1, for further analysis. Our results showed that miR-214 precursor transfection significantly reduced the activity of a luciferase reporter gene fused to the CSF1 3′-UTR, with 29.60% and 30.61% reduction, compared to the negative control groups (Figure 6B, *P* = 0.0227 and 0.0093, respectively, for MKN45 and BGC823 cell lines). Whereas when miR-214 inhibitor was transfected, luciferase activity was increased by 34.49% and 42.25% in MKN45 and BGC823 cells compared with the controls (Figure 6C, *P* = 0.0115 and 0.0085, respectively).

We further determined the expression of CSF1 protein by western blot in GC cells transfected with miR-214 precursor or inhibitor. Consistent with the luciferase results, the expression of CSF1 protein was significantly decreased in SGC7901 and MKN45 cells (Figure 6D-E, *P* = 0.0037 and 0.0066, respectively) transfected with miR-214 precursor and increased in MKN28 and BGC823 cells (Figure S7A-B, *P* = 0.0049 and 0.0416, respectively) transfected with miR-214 inhibitor, compared with the respective controls. These data suggested that miR-214 inhibits CSF1 translation in gastric cancer cells.

## Discussion

Emerging evidence has highlighted the crucial impact of miRNA dysregulation on tumourigenesis of human carcinomas [3], [4]. MiRNA dysregulation promotes cell proliferation, confers resistance to apoptosis, and enhances invasiveness and metastasis, through repressing the downstream tumour suppressors or inducing the accumulation of target oncogenes, and thus is involved in the initiation, progression, and metastasis of human tumours.

Among the plethora of miRNAs, dysregulation of miR-214 was found in plenty of human cancers [15]–[22]. Downregulation of miR-214 in cervical cancer has been reported by several groups, and miR-214 has been shown to inhibit the growth, migration, and invasion of cervical cancer cells by targeting oncogenes MEK3, JNK1, Plexin-B1, and GALNT7 [15]–[17]. MiR-214 has also been found to be decreased in breast cancer and contribute to breast tumourigenesis by allowing aberrantly elevated oncogene Ezh2 accumulation and subsequent unchecked cell proliferation and invasion [18]. However, Penna et al. have shown that miR-214 is highly expressed in human melanomas and contributes to melanoma tumour progression through suppression of TFAP2C, a homologue of a well-established melanoma tumour suppressor [19]. These results indicate that the role of miR-214 in cancer is tissue- or cell- specific.

A recent study by Ueda et al. showed that dysregulation of miR-214 was correlated with clinical stage, peritoneal dissemination and survival of patients [20]. However, the exact role of miR-214 in the progression of gastric carcinoma and its underlying molecular mechanism remains elusive. Here, we show that miR-214 was substantially reduced in GC, especially in metastatic tissues, suggesting a potential role of miR-214 in predicting lymph node metastasis, which was further supported by our ROC curve analysis. In addition, we found that low expression of miR-214 had an inclination towards a larger tumour size. To address whether the dysregulation of miR-214 bears a biological significance, we utilized a follow-up study and functional analysis of miR-214 *in vitro*. Nonetheless, our data suggest that miR-214 has no effect on patient outcome, including relapse status and survival. Our data showed that overexpression of miR-214 with lentivirus miR-214-expressing vector dramatically reduced migration and invasion of GC cell lines and knockdown of miR-214 could facilitate cell proliferation, apoptosis, migration and invasion in a cell-specific manner, with no influence on cell apoptosis.

CSF1 (colony stimulating factor 1 (macrophage)) is a critical hematopoietic growth factor involving in macrophage cell differentiation, proliferation and activation and it is also present in non-hematopoietic cells [21]. CSF-1 affects cellular survival and proliferation via binding to its receptor encoded by the c-fms gene [22]. Previous studies showed that CSF1 exerted important roles in promoting tumor angiogenesis in leiomyosarcoma [23] and Lewis lung carcinoma cells via VEGF induction [24]. Aligeti S et al. suggested that CSF1 has positive effect of on the attachment to pleural mesothelial cells, invasion and proliferation of endometrial epithelial cells [25]. And elevated serum concentrations of has been correlated with disease stage, lymph node metastasis and poor prognosis in colorectal cancer patients [26]. Here we identified CSF1 was a direct target of miR-214 in gastric cancer through luciferase assays and further confirmed by western blot analysis. Our data suggested that downregulation of miR-214 may facilitate the proliferation, migration and invasion capacity of gastric cancer cell through the CSF1-mediated signal pathway.

In the previous study, Ueda et al. showed that relatively higher expression of miR-214 in GC was associated with unfavorable overall survival in 101 patients (obtained from the University of Tokyo and Hiroshima University) [20], however, our data indicated that miR-214 was not significantly correlated with patient outcome. We assume that this discrepancy may be due to the different miRNA expression detection methods, sample sizes, and follow-up times between their research and ours. Ueda et al. divided the samples into two classes (high and low expression) using the mean levels of miRNA expression measured on a microRNA microarray as a threshold, while our division of high and low expression groups was based on the median level of expression gained from the qPCR results. Intriguingly, in previous studies, miR-214 expression has been shown to be upregulated in ovarian cancer compared with normal ovarian cells by Yang et al. [27], whereas opposite results were reported by Nam et al. [28]. Both of these two groups used miRNA microarrays to examine the miRNA expression. Thus, we cannot exclude the possibility that microarray analysis may not be as sensitive and accurate in analyzing the expression profiles of a specific miRNA. As for the effect of miR-214 on the prognosis of patients with GC, partially consistent with the results of Ueda et al., we also found a tendency that high expression of miR-214 was unfavorable for survival when the follow-up time was longer than 40 months (hazard ratio (HR)  = 1.19 for overall survival, HR = 1.23 for relapse-free survival), as shown in Figure 2, although the trend was not statistically significant. In any case, further investigation with a larger cohort and different experimental settings are needed to resolve this issue.

As for the discordance of miR-214 lentivirus vectors and precursor transfection assays, we conjecture it is a specific consequence of the biological activities of miR-214. A moderate and stable expression rather than tens of thousands of non-physiopathological overexpresion may help miR-214 function well.

Taken together, we demonstrated that miR-214 was substantially reduced in GC, especially in metastatic tissues, suggesting a potential role of miR-214 in predicting lymph node metastasis. Our ROC curve analysis showed the relatively high specificity and sensitivity of miR-214 for discriminating GC cases from nontumourous controls, and separating patients with and without lymph node metastasis, thus prompting its potential utility as a novel biomarker for GC and lymph node metastasis, which will be helpful to facilitate the clinical management of GC. However, our data indicated that miR-214 has no effect on patient prognosis, indicating that miR-214 alone is not enough to affect patient survival. Our data showed that miR-214 could inhibit cell proliferation, migration and invasion in a cell-specific manner, with no influence on cell apoptosis. To explore a direct targeting mechanism, we identified CSF1 as a direct target of miR-214. Conclusively, our data indicated that downregulation of miR-214 may contribute to GC progression by inducing aberrantly elevated CSF1 accumulation and subsequently promoting the proliferation, migration, and invasion of GC cells.

## Supporting Information

Figure S1 **Evaluation of miR-214 as a novel biomarker for gastric cancer and lymph node metastasis.** (A) The ROC curves of miR-214 reflected strong separation between gastric cancer tissues and nontumourous tissues, with an area under curve (AUC) of 0.7764 (95% CI, 0.6466–0.9062). (B) To test the ability of miR-214 in GC as a biomarker for lymph node metastasis, ROC curves were established. We observed clear separations between the patients with and without lymph node metastasis, with an AUC of 0.5880 (95% CI, 0.4526–0.7166).
(TIF)

Figure S2 **Effect of miR-214 precursor on the cell biological behavior of SGC7901 and MKN45 cells.** (A, B) The expression of miR-214 was detected by RT-qPCR in cells transfected with miR-214 precursor, NC and nontransfected (mock) groups. Fold-change of miR-214 expression was calculated, which means relative miR-214 expression in miR-214-transfected group and NC group compared to that in the nontransfected group. MiR-214 precursor significantly enhanced miR-214 level in SGC7901 and MKN45 cells (^*^ *P*<0.05). (C-N) MiR-214 precursor demonstrated no effect on the cell proliferation, migration, invasion or apoptosis of SGC7901 and MKN45 cell lines (*P*>0.05).
(TIF)

Figure S3 **Transfection efficiency monitored by RT-qPCR.** (A, B) Representative profiles of cells transfected with lentivirus miR-214-expressing vector in SGC7901 and MKN45 cells (magnification 100×) after puromycin selection. We monitored the GFP expression for 4 weeks and the results showed that 80%–90% of the cells in the visual field expressed the GFP marker protein. (C, D) MiR-214-expressing vector significantly increased miR-214 level in SGC7901 and MKN45 cells, compared with the LV3NC treated cells (^*^ *P*<0.05). (E, F) MiR-214 inhibitor led to a dramatic decrease of miR-214 level in MKN28 and BGC823 cells (^*^ *P*<0.05).
(TIF)

Figure S4 **Influence of miR-214 inhibitor on the proliferation, migration and invasion of GES-1 cells.** (A) MiR-214 inhibitor significantly reduced miR-214 expression in GES-1 cells (^*^ *P*<0.05). (B, C) Downregualtion of miR-214 with miR-214 inhibitor could enhance the proliferation of GES-1 cell line (*P* = 0.0474). (D, E) MiR-214 inhibitor significantly promote cell invasion of GES-1 cells (*P* = 0.0046). And our data showed a pro-migration tendency of miR-214 inhibitor in GES-1 cell line (*P* = 0.0879).
(TIF)

Figure S5 **Effect of miR-214 on cell proliferation in MKN45 and MKN28 cells.** (A, C) Representative profiles after transfection with lentivirus miR-214-expressing vector in MKN45 and miR-214 inhibitor in MKN28 cells (magnification 100×). (B, D) The data showed that LV3-hsa-miR-214 and miR-214 inhibitor transfection could not influence cell proliferation ability of MKN45 and MKN28 cells (P = 0.0726 and 0.0938, respectively).
(TIF)

Figure S6 **MiR-214 reduced cell migration and invasion ability in MKN45 cells.** (A, C) Representative photographs of migration and invasion assays in MKN45 cells (magnification 100×) are shown. (B, D) MiR-214-expressing lentivirus vector transfection led to a pronounced decrease of the migration and invasion ability in MKN45 cell line (*P* = 0.0172 and 0.0143, respectively).
(TIF)

Figure S7 **Silencing miR-214 with miR-214 inhibitor could increase the expression of CSF1 protein.** (A) Expression of CSF1 protein in miR-214 inhibitor-transfected and inhibitor NC treated cells was analyzed by western blot. (B) Downregulation of miR-214 significantly elevated the level of CSF1 in MKN28 (*P* = 0.0049) and BGC823 cells (*P* = 0.0416).
(TIF)

Table S1 **PCR primers for the 3′-UTR fragments of miR-214 target genes.**
(DOC)
