# Supplementary figures and images for: Clinicopathological Significance of MicroRNA-214 in Gastric Cancer and Its Effect on Cell Biological Behaviour
Source: PLoS One. 2014 Mar 10;9(3):e91307. doi: 10.1371/journal.pone.0091307 (PMC3948864; doi:10.1371/journal.pone.0091307)

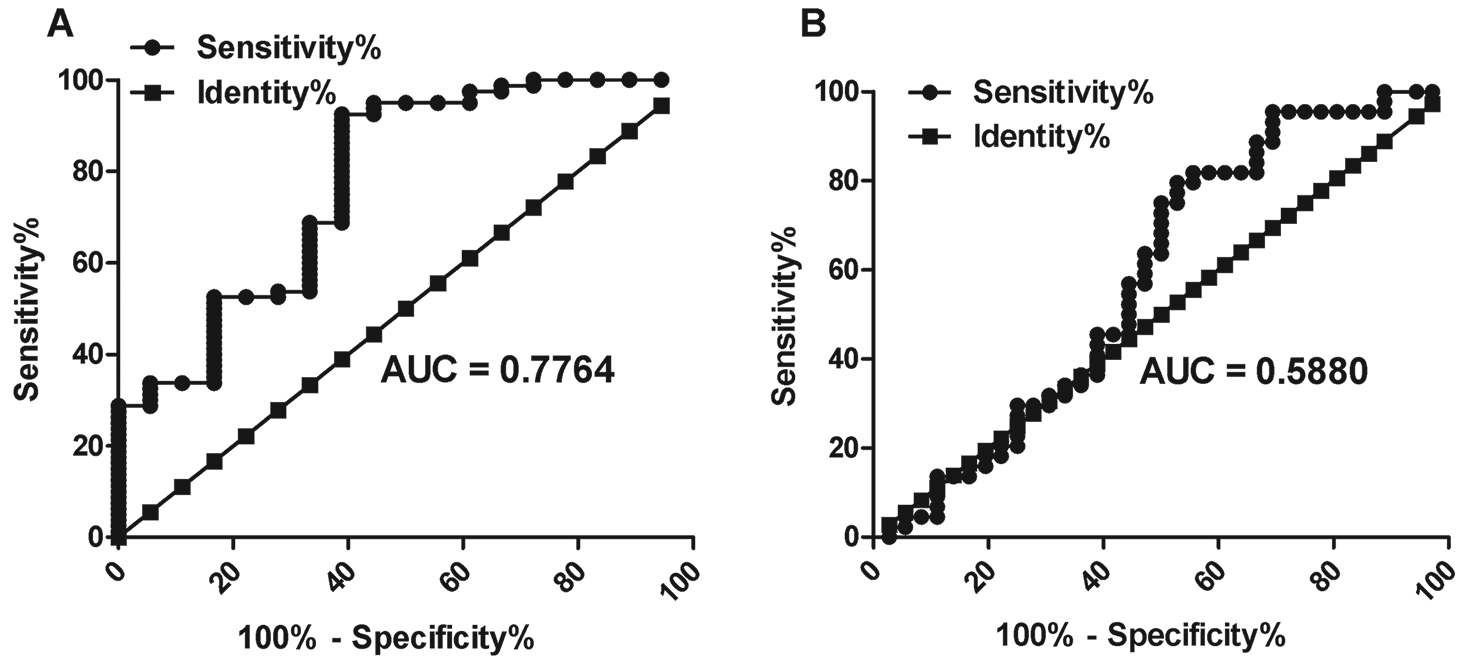

Supplement: Figure S1 — Evaluation of miR-214 as a novel biomarker for gastric cancer and lymph node metastasis. (A) The ROC curves of miR-214 reflected strong separation between gastric cancer tissues and nontumourous tissues, with an area under curve (AUC) of 0.7764 (95% CI, 0.6466–0.9062). (B) To test the ability of miR-214 in GC as a biomarker for lymph node metastasis, ROC curves were established. We observed clear separations between the patients with and without lymph node metastasis, with an AUC of 0.5880 (95% CI, 0.4526–0.7166). (TIF) [file pone.0091307.s001.tif]

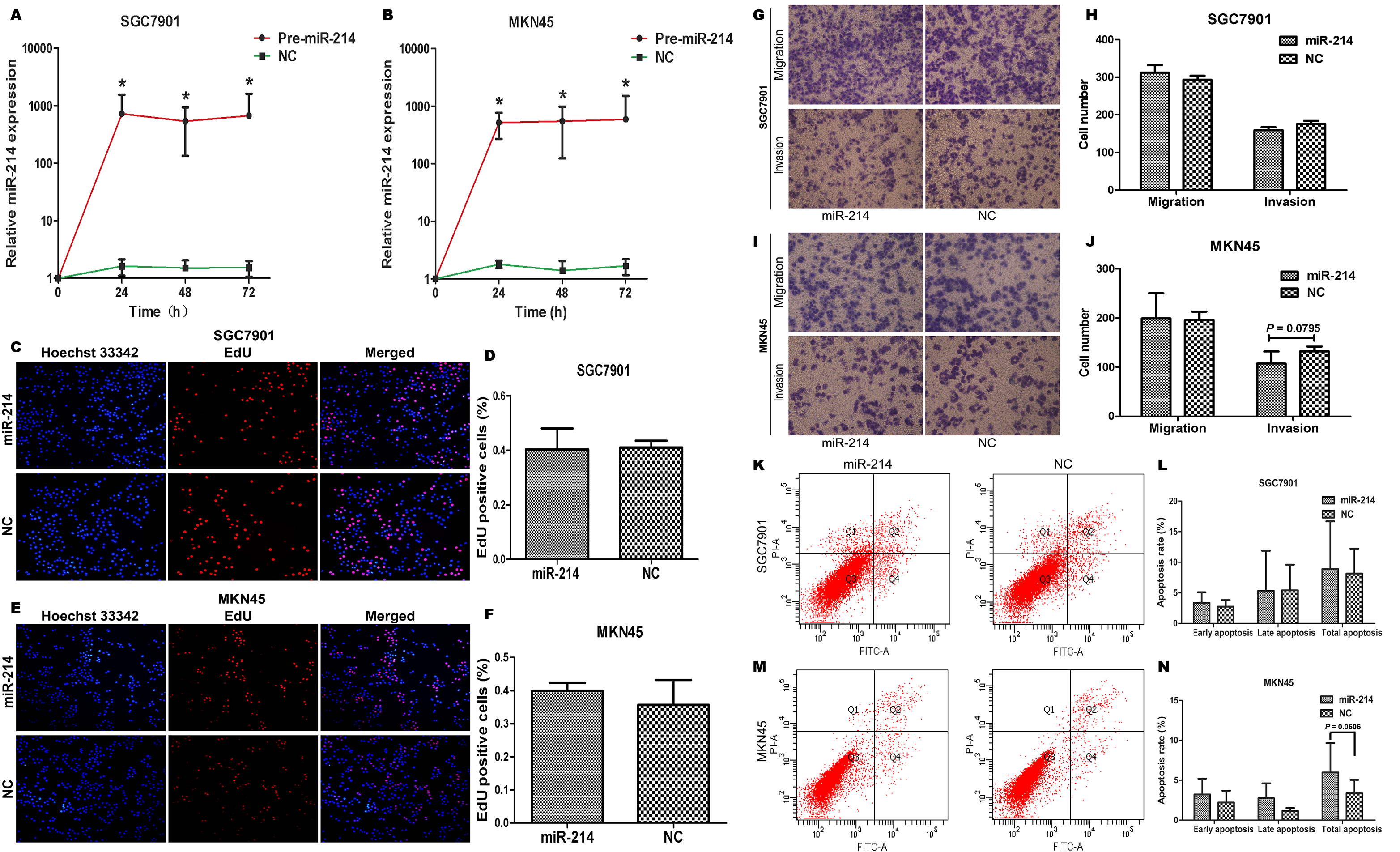

Supplement: Figure S2 — Effect of miR-214 precursor on the cell biological behavior of SGC7901 and MKN45 cells. (A, B) The expression of miR-214 was detected by RT-qPCR in cells transfected with miR-214 precursor, NC and nontransfected (mock) groups. Fold-change of miR-214 expression was calculated, which means relative miR-214 expression in miR-214-transfected group and NC group compared to that in the nontransfected group. MiR-214 precursor significantly enhanced miR-214 level in SGC7901 and MKN45 cells (* P<0.05). (C-N) MiR-214 precursor demonstrated no effect on the cell proliferation, migration, invasion or apoptosis of SGC7901 and MKN45 cell lines (P>0.05). (TIF) [file pone.0091307.s002.tif]

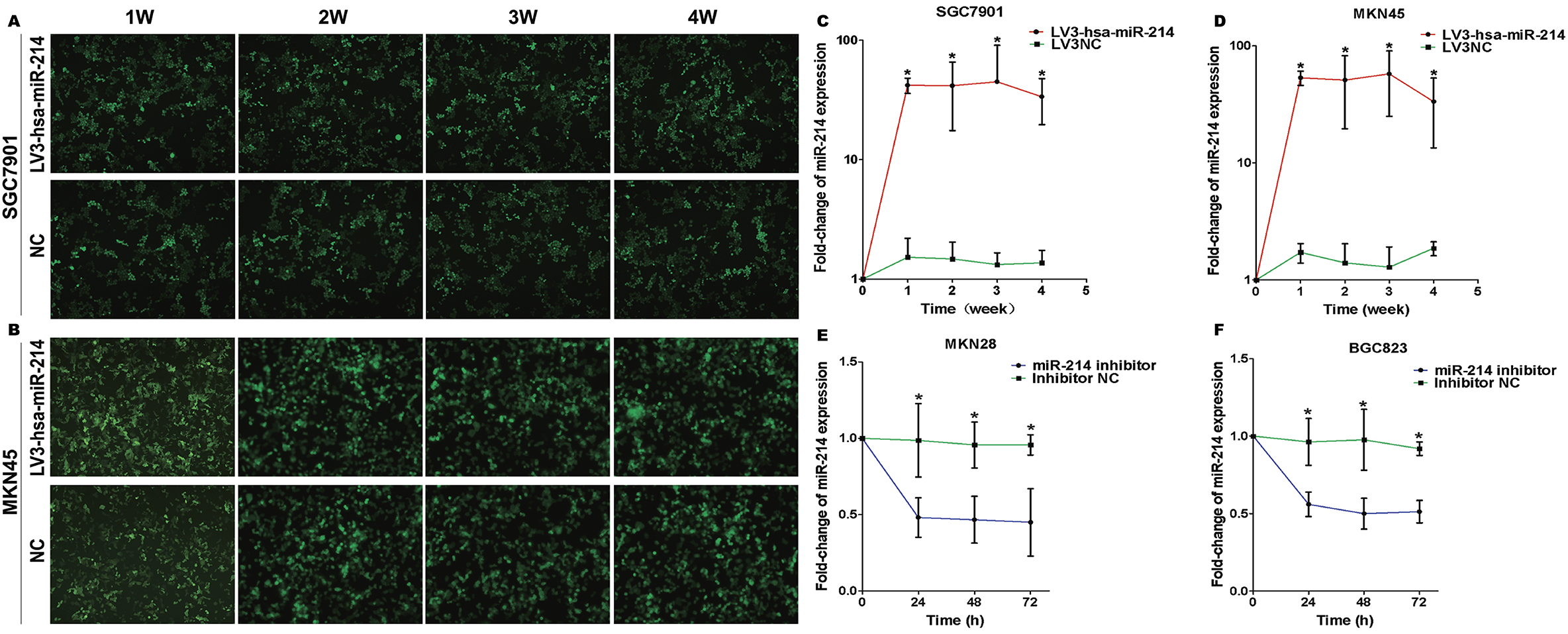

Supplement: Figure S3 — Transfection efficiency monitored by RT-qPCR. (A, B) Representative profiles of cells transfected with lentivirus miR-214-expressing vector in SGC7901 and MKN45 cells (magnification 100×) after puromycin selection. We monitored the GFP expression for 4 weeks and the results showed that 80%–90% of the cells in the visual field expressed the GFP marker protein. (C, D) MiR-214-expressing vector significantly increased miR-214 level in SGC7901 and MKN45 cells, compared with the LV3NC treated cells (* P<0.05). (E, F) MiR-214 inhibitor led to a dramatic decrease of miR-214 level in MKN28 and BGC823 cells (* P<0.05). (TIF) [file pone.0091307.s003.tif]

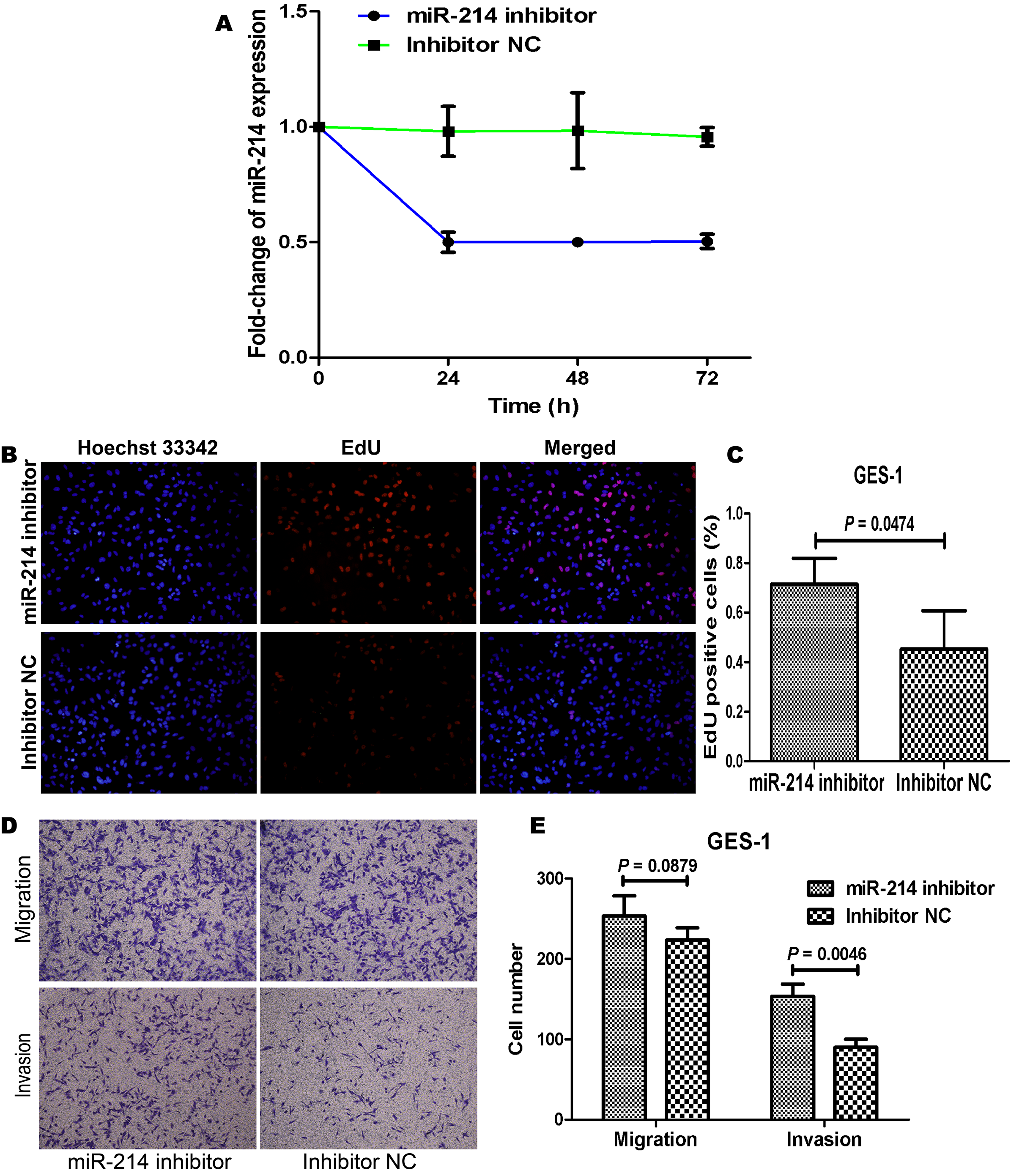

Supplement: Figure S4 — Influence of miR-214 inhibitor on the proliferation, migration and invasion of GES-1 cells. (A) MiR-214 inhibitor significantly reduced miR-214 expression in GES-1 cells (* P<0.05). (B, C) Downregualtion of miR-214 with miR-214 inhibitor could enhance the proliferation of GES-1 cell line (P = 0.0474). (D, E) MiR-214 inhibitor significantly promote cell invasion of GES-1 cells (P = 0.0046). And our data showed a pro-migration tendency of miR-214 inhibitor in GES-1 cell line (P = 0.0879). (TIF) [file pone.0091307.s004.tif]

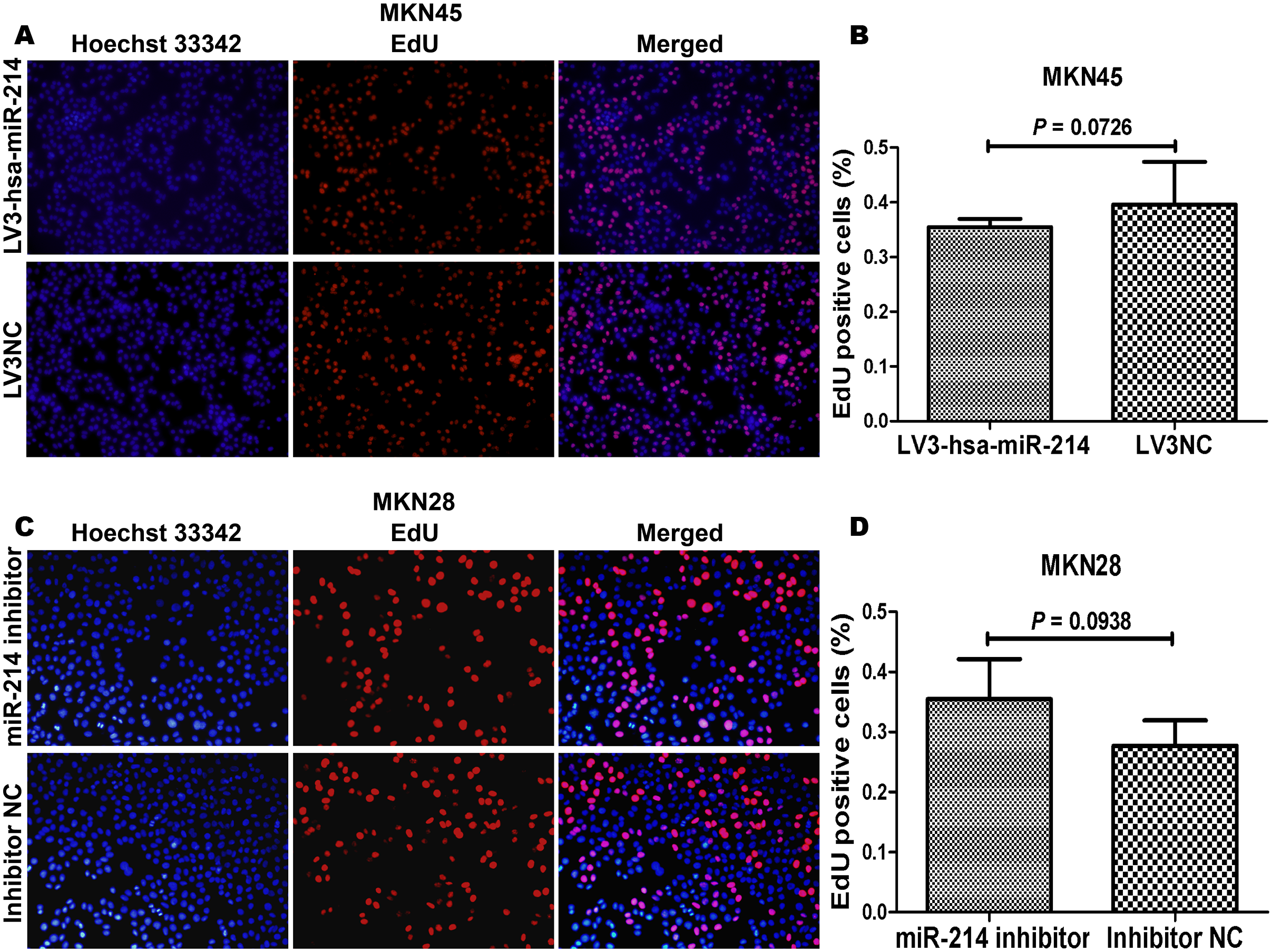

Supplement: Figure S5 — Effect of miR-214 on cell proliferation in MKN45 and MKN28 cells. (A, C) Representative profiles after transfection with lentivirus miR-214-expressing vector in MKN45 and miR-214 inhibitor in MKN28 cells (magnification 100×). (B, D) The data showed that LV3-hsa-miR-214 and miR-214 inhibitor transfection could not influence cell proliferation ability of MKN45 and MKN28 cells (P = 0.0726 and 0.0938, respectively). (TIF) [file pone.0091307.s005.tif]

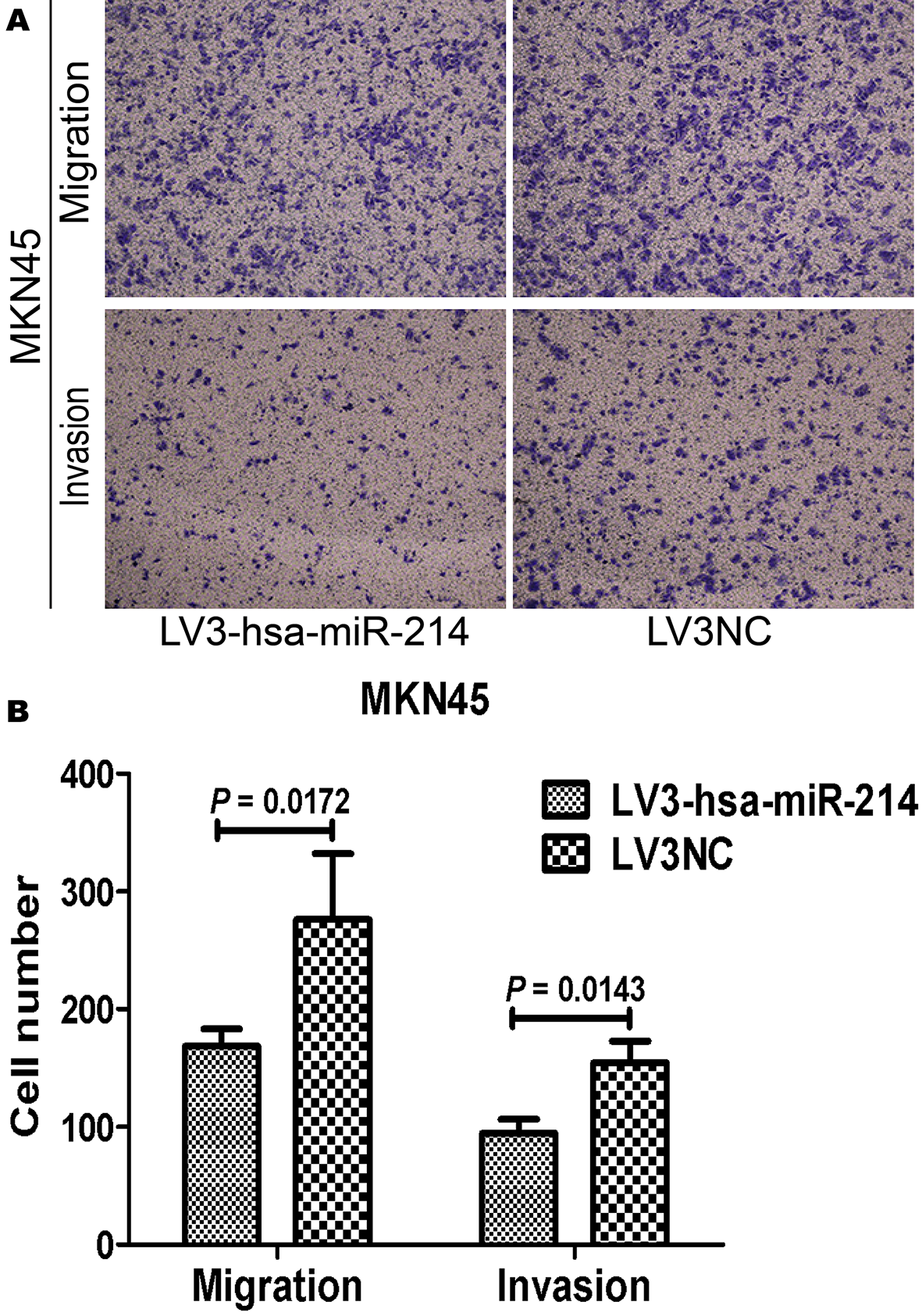

Supplement: Figure S6 — MiR-214 reduced cell migration and invasion ability in MKN45 cells. (A, C) Representative photographs of migration and invasion assays in MKN45 cells (magnification 100×) are shown. (B, D) MiR-214-expressing lentivirus vector transfection led to a pronounced decrease of the migration and invasion ability in MKN45 cell line (P = 0.0172 and 0.0143, respectively). (TIF) [file pone.0091307.s006.tif]

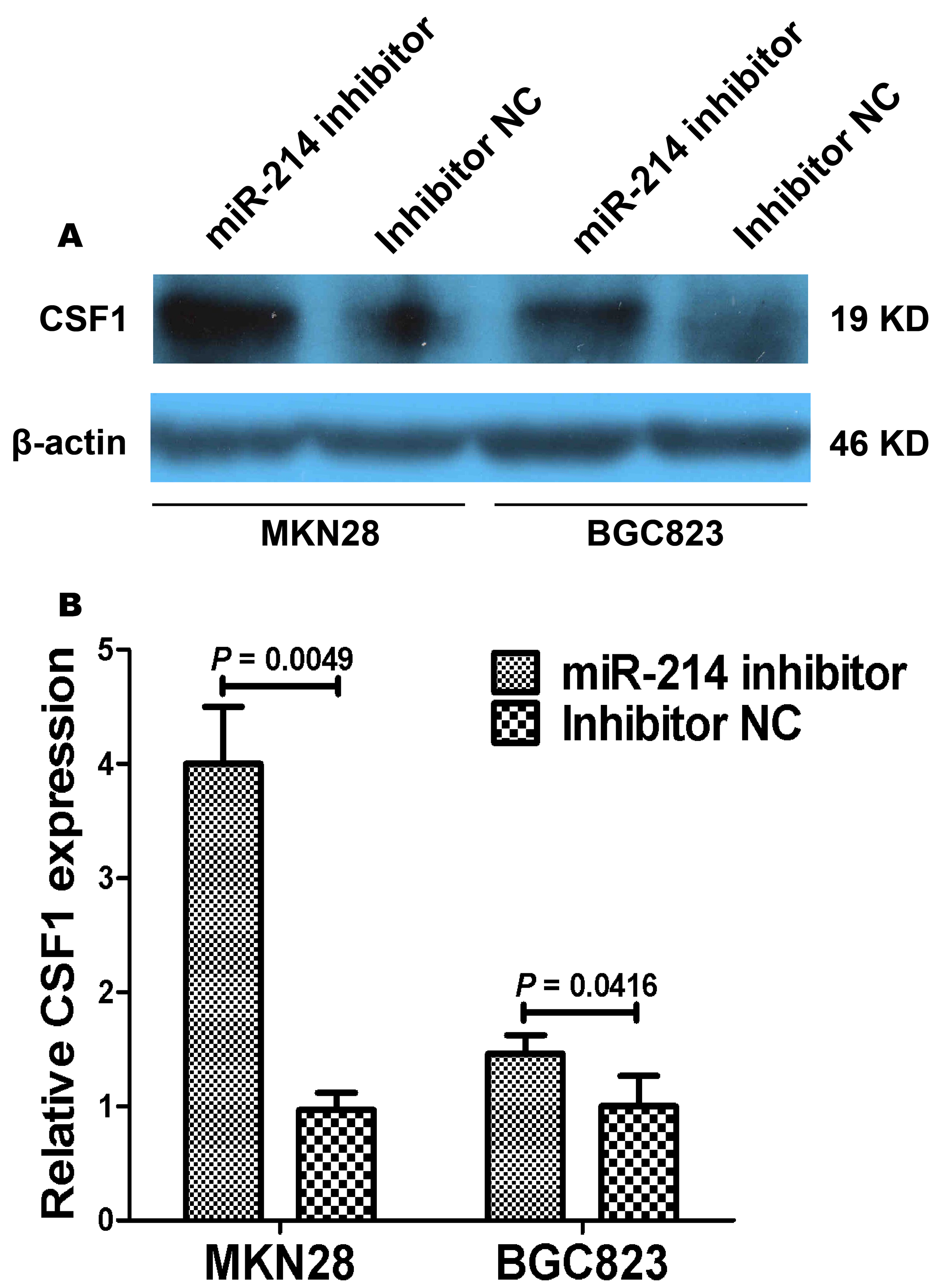

Supplement: Figure S7 — Silencing miR-214 with miR-214 inhibitor could increase the expression of CSF1 protein. (A) Expression of CSF1 protein in miR-214 inhibitor-transfected and inhibitor NC treated cells was analyzed by western blot. (B) Downregulation of miR-214 significantly elevated the level of CSF1 in MKN28 (P = 0.0049) and BGC823 cells (P = 0.0416). (TIF) [file pone.0091307.s007.tif]
