# Supplementary material for: Clinicopathological Significance of MicroRNA-214 in Gastric Cancer and Its Effect on Cell Biological Behaviour
Source: PLoS One. 2014 Mar 10;9(3):e91307. doi: 10.1371/journal.pone.0091307 (PMC3948864; doi:10.1371/journal.pone.0091307)
Supplement: Table S1 — PCR primers for the 3′-UTR fragments of miR-214 target genes. (DOC) [file pone.0091307.s008.doc]

| **Table S1** |  |
| --- | --- |
| **PCR primers for the 3'-UTR fragments of miR-214 target genes** | |
| **Definition** | **Sequence** |
| NOTCH2-F | gcggagctcCACCTCCAGTGTAGAGACATAAC |
| NOTCH2-R | gcgtctagaCAGGACGCTAGTGTAGAATCTTC |
| FGFR1-F | gcggagctcTCTTCCTGTCCAAACTCCATCC |
| FGFR1-R | gcgtctagaTTACAATAGTCGCCAACACTGC |
| CSF1-F | gcggagctcAGCCTGAGAGACGGGAAGAG |
| CSF1-R | gcgtctagaGAGTGGAGGACGGACGGATG |
| AGAP2-F | gcggagctcCCAGAGGGAGGGTTTAGTAC |
| AGAP2-R | gcgtctagaGGAACAAAGCAATAAATTACAAGG |
| CREB1-F | gcggagctcTGAAGATTTAAGTGTTAATTGCTG |
| CREB1-R | gcgtctagaTTGTGACTTTGCTTGATTTCTG |
